# An Unusual Case of an Obstructed Inguinal Hernia Sac Containing Testis in an Adult Male

**DOI:** 10.7759/cureus.87952

**Published:** 2025-07-14

**Authors:** Apoorva B Patil, Raghupathy Thirunavikkarasu, Sairam KR

**Affiliations:** 1 General Surgery, Sree Balaji Medical College and Hospital, Chennai, IND

**Keywords:** adult male, hernia sac contents, inguinal hernia, obstructed inguinal hernia, testis

## Abstract

Inguinal hernia can be defined as the protrusion of the abdominal contents through the inguinal canal. It might be observed as a post-surgical complication. Hernias can be classified as either direct or indirect, each with distinct underlying etiologies and can be noted in all age groups with no particular demarcation. Inguinal hernias are common, but the presence of testis within the hernial sac in adults is a rare occurrence. This case reports a 28-year-old male presenting with severe pain in the inguinal region for the last two days. The pain was dull-aching and progressive in nature. The patient was diagnosed with an obstructed right inguinal hernia, which had progressively increased in size. He underwent successful management through Lichtenstein tension-free mesh repair surgery.

## Introduction

Inguinal hernias are classified into two types, direct and indirect. The majority of the herniae have a sac of peritoneum into which any ambulatory intraperitoneal structure can herniate. Underlying etiology is defined as weakened or disruption of abdominal muscles, caused by congenital connective tissue abnormalities, pharmacologic effects like chronic glucocorticoid administration, chronic smoking, chronic cough or constipation, and old age [1,2]. Contents of the hernial sac are usually bowel, omentum, and in rare circumstances, testis, urinary bladder or even the appendix. When the hernial sac becomes irreducible, it is called an obstructed hernia [3]. The neck of a peritoneal loculus inside the sac, or the fascial defect’s boundaries, could be the source of the constriction and might cause excruciating discomfort in the abdomen, nausea, vomiting, and difficulty passing gas or having a bowel movement [4]. The presence of unusual organs in the hernial sac could create a tricky situation for the operating surgeon. This is the case report of a 28-year-old male with a major complaint of severe pain in the inguinal region. He was managed by emergency Lichtenstein tension-free mesh repair.

## Case presentation

A 28-year-old male patient of South-Asian ethnicity was examined in the emergency department with severe right-sided inguinal region pain for a period of two days. The patient reported experiencing intense throbbing right-sided inguinal region pain for the past two days. The pain initially began as a dull aching pain one week ago and progressively worsened in severity. The patient gave a history of having right groin swelling for approximately three years. It was insidious in onset and gradually progressive. The swelling initially measured approximately 3 × 4 cm and gradually increased in size to 10 × 7 cm. It was previously spontaneously reducible upon lying down; however, over the past 48 hours, from the time of reporting, it had become irreducible.

The patient denied any history of constipation, loose stools, altered bowel habits, vomiting, fever episodes, or any urinary complaints. There was no history of undescended testis previously. The testis could not be palpated separately due to the complete hernia. The patient’s professional background included experience in construction, specifically masonry. Hence, the patient was consistently engaged in strenuous activities that required lifting heavy weights. He also had a history of smoking, having commenced the habit at an early age, and smoking almost one packet of cigarettes per day. The patient was well-built and nourished and did not show any signs of dehydration.

**Figure 1:**
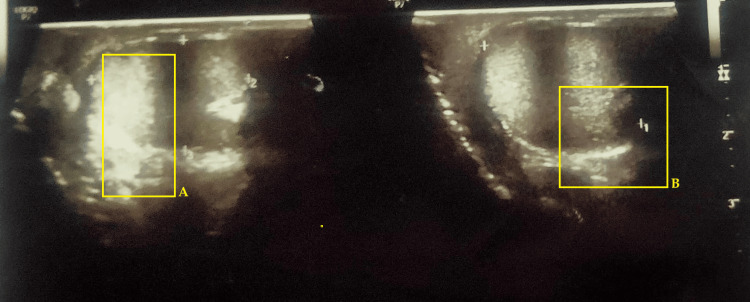
Ultrasound images of the hernial sac protruding through a defect in the anterior abdominal wall. A: right testis, B: left testis; testis not clearly visualized, tubular, mixed echogenic structures above/near testis noted as a classic for bowel in hernia sac.

On clinical examination, a pyriform-shaped swelling of size 12 x 8 centimetres was present over the right inguinal region. The swelling extended from the mid-inguinal point to the base of the scrotum. It was tense, firm in consistency, and tender on palpation. The swelling was irreducible. The patient underwent a thorough clinical and radiological assessment, and all necessary baseline blood investigations were conducted. Ultrasound of the abdomen and pelvis revealed a defect in the right inguinal region, with bowel loops and omentum as its contents, extending into the right scrotal sac (Figure 1).

**Figure 2:**
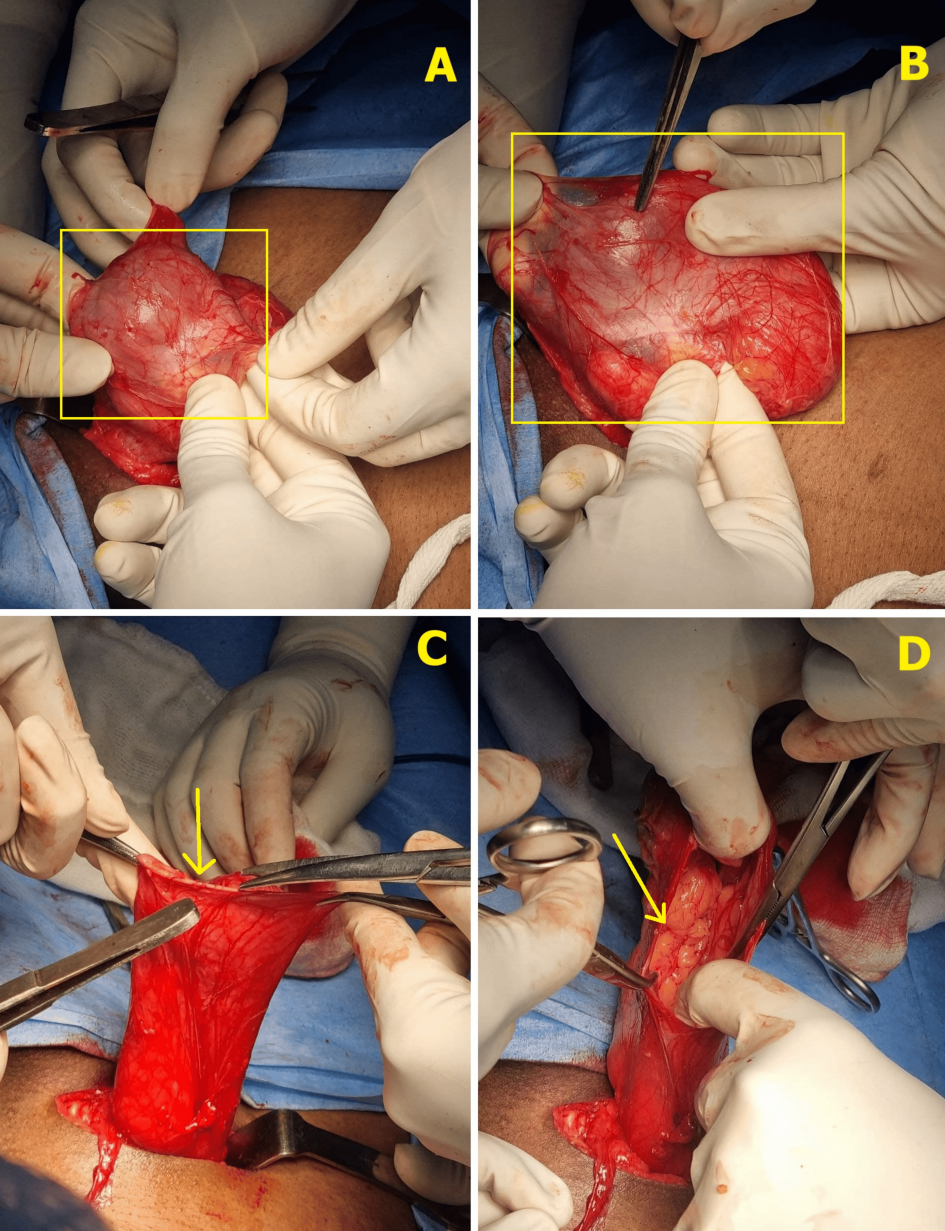
Intraoperative images of the patient showing testis in the hernia sac. A, B: indirect hernia sac, C, D: dissected sac showing one of its contents-omentum.

**Figure 3:**
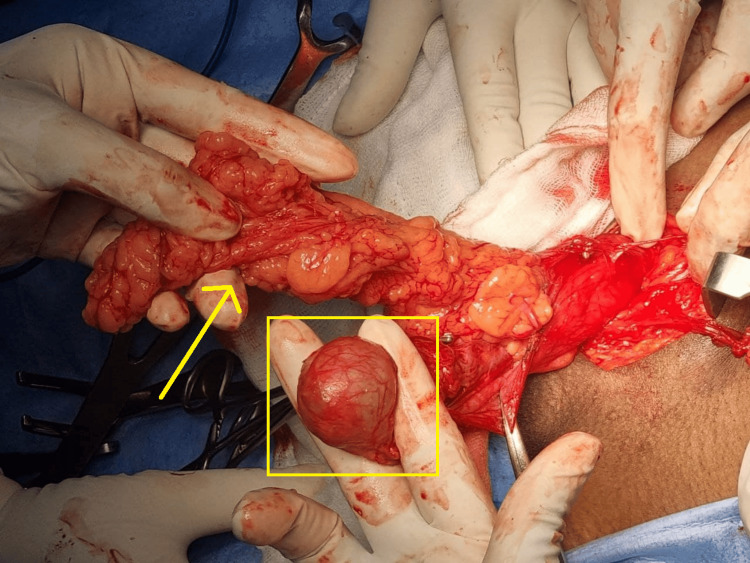
Indirect hernia sac showing the contents of the sac along with the testis.

Herniated bowel showed sluggish peristalsis and vascularity on color doppler, suggestive of obstructed inguinoscrotal hernia. The patient was scheduled to undergo an emergency Lichtenstein tension-free mesh repair surgery. The patient was subjected to general anesthesia, and an oblique incision was made two-finger breadth inferior and medial to the anterior superior iliac spine and extended medially 6 centimetres under standard operative conditions. The incision was deepened, and the sac was identified. Intraoperative findings showed a hernial sac extending from the deep ring through the superficial ring till the base of the scrotum and was identified to be lateral to the inferior epigastric artery. The sac contained omentum and testis with thinned-out vas deferens (undescended testis) (Figures 2, 3).

The omentum was pushed back into the peritoneum, and the sac was transfixed and ligated. Lytle’s repair was completed with the narrowing of the deep ring. The testis was separated from the sac, and orchidectomy was carried out. The Lichtenstein tension-free herniorrhaphy was completed with the use of a propylene mesh. The patient had a smooth recovery after surgery and was discharged on the third postoperative day with advice of 15, 30, 60 and 90 days follow-up. 

## Discussion

Indirect hernia is the most common groin hernia in both males and females, with an incidence of 90% and 10% respectively [2]. Inguinal hernia is common after surgical procedures. It is also reported to be the most common surgery carried out by surgeons [2,3]. Though inguinal hernia is common, the contents of the hernia sac, such as urinary bladder, colon, cecum, appendix and others, can be presented as a surgical challenge [3]. It can have varied clinical presentations. In present times, inguinal hernia is frequently diagnosed early due to the presence of pain or swelling in the groin region. It is commonly observed as a bulge in the groin area, which usually enlarges over time [5]. This was also an observation in this patient, with the swelling noted in his right groin area for the last 3 years. Inguinal hernia can be diagnosed with the help of radiological modalities such as ultrasound, computed tomography and magnetic resonance imaging [2,5]. Testicular descent is an intricate process influenced by various factors such as gonadal ligament growth and reorganisation, hormones, and interplay between different species [6]. Although the inception of the testes is in the abdomen, they migrate to the scrotum, making them susceptible to pathological conditions [7]. Crucial functions in the descending process are performed by hormones such as calcitonin gene-related peptide (CGRP), testosterone, anti-Müllerian hormone, and insulin-like growth hormone 3 (INSL3). Numerous structural abnormalities, including undescended testicles, hydrocele, and indirect inguinal hernias, can be caused by a deviation or anomaly in this process [8]. The hernial sac in this case went through a deep ring and contained the testis, omentum, and a thinned-out vas deferens, which was also a common observation in similar cases [9,10]. There is an evidence-based recommendation to monitor asymptomatic inguinal hernia in males, though most of the patients from randomised trials have been reported to undergo subsequent surgery due to pain [11]. The testis in the hernial sac was a rare incidental finding, highlighting various types of hernias. This case report underscores unexpected findings that may be encountered during surgical exploration and the need for a tailored management approach.

## Conclusions

The presence of testis within the hernia sac is a rare clinical finding. This case highlights the importance of such unusual clinical presentations in patients with indirect inguinal hernia in adults. The management of such cases should be planned meticulously with a focus on preserving testicular function and its placement. More such cases need to be reported to create awareness of this rare presentation in adults.
